# The role of transcription factor FOXA1/C2/M1/O3/P1/Q1 in breast cancer

**DOI:** 10.1097/MD.0000000000037709

**Published:** 2024-04-12

**Authors:** Hui Yuan, Yu Liang, Shaorun Hu, Jinxiang Chen, Jingcan You, Jun Jiang, Mao Luo, Min Zeng

**Affiliations:** a Department of Pharmacy, The Affiliated Hospital, Southwest Medical University, Luzhou, Sichuan, China; b Basic Medicine Research Innovation Center for Cardiometabolic Diseases, Ministry of Education, Southwest Medical University, Luzhou, Sichuan, China; c Integrated Traditional Chinese and Western Medicine, Affiliated Hospital of Traditional Chinese Medicine, Southwest Medical University, Luzhou, Sichuan, China; d Laboratory for Cardiovascular Pharmacology, Department of Pharmacology, School of Pharmacy, Southwest Medical University, Luzhou, Sichuan, China; e Municipal Key Laboratory of Thrombosis and Vascular Biology, Luzhou, Sichuan, China; f Department of General Surgery (Thyroid Surgery), The Affiliated Hospital, Southwest Medical University, Luzhou, Sichuan, China; g Metabolic Vascular Diseases Key Laboratory of Sichuan Province, Luzhou, Sichuan, China.

**Keywords:** breast cancer, FOX transcription factor, immune infiltration, mRNA, prognosis

## Abstract

Breast cancer is a common malignancy with the highest mortality rate among women worldwide. Its incidence is on the rise year after year, accounting for more than one-tenth of new cancers worldwide. Increasing evidence suggests that forkhead box (FOX) transcription factors play an important role in the occurrence and development of breast cancer. However, little is known about the relationship between the expression, prognostic value, function, and immune infiltration of FOX transcription factors in tumor microenvironment. We used bioinformatics to investigate expression and function of FOX factor in breast cancer. Our results revealed the expression levels of FOXA1 and FOXM1 were significantly higher in breast cancer tissues than in normal tissues. The high expression of mRNA in FOXA1 (*P* < .05), FOXM1 (*P* < .01), and FOXP1 (*P* < .05) groups was related to tumor stage. Survival analysis results showed that increased FOXP1 mRNA levels were significantly associated with overall survival (OS), recurrence-free survival (RFS), and distant metastasis-free survival (DMFS) in all patients with breast cancer (*P* < .05). Patients with the FOXA1 high-expression group had better RFS and DMFS than the low-expression group (*P* < .05), while patients with FOXM1 high-expression group had worse RFS, OS, and DMFS than the low-expression group (*P* < .05). Meanwhile, mutation analysis showed that genetic alterations in FOX transcription factors were significantly associated with shorter OS and progression-free survival (*P* < .05), but not with disease-free survival (*P* = .710) in patients with breast cancer. FOXP1, FOXA1, and FOXM1 may be used as potential biomarkers to predict the prognosis of patients with breast cancer. Functional enrichment indicated that FOX was mainly involved in cell division, cell senescence, cell cycle, and prolactin signaling pathway. In patients with breast cancer, *FOXC2* expression was negatively correlated with the infiltration of B cells and positively correlated with the infiltration of neutrophils and dendritic cells. However, FOXM1 was negatively correlated with the infiltration of CD8 + T cells and macrophages and positively correlated with the infiltration of neutrophils and dendritic cells. These findings provided novel insights into the screening of prognostic biomarkers of the FOX family in breast cancer and laid a foundation for further research on the immune infiltration of the FOX transcription factor family members in tumors.

## 1. Introduction

Breast cancer (BRCA) is a common malignancy with the highest mortality rate among women worldwide.^[1]^ More than 1.5 million women worldwide (25% of all women with cancer) are diagnosed with BRCA each year, and the incidence is on the rise.^[2]^ Surgery is the primary treatment for BRCA, but with the continuous development of diagnosis and systemic comprehensive treatment, the diagnosis and treatment of BRCA are changing.^[3]^ In 2000, Perou et al^[4]^ described 4 basic molecular subtypes of BRCA: luminal A, luminal B, HER2-enriched, and basal-like. Despite advances in cancer treatment in recent decades, current evidence-based medicine suggests that progress against BRCA has been slow over the past decade.^[5]^ BRCA is a highly heterogeneous disease. Different molecular subtypes have different clinical significance, and the current biomarkers related to prognosis have certain limitations.^[6]^ Therefore, exploring to understand the pathogenesis of BRCA at the molecular level and finding biomarkers as prognostic indicators are important to effectively improve prognosis and individualize treatment.

The forkhead box (FOX) is an evolutionarily conserved superfamily of transcription factors with an evolutionarily conserved “forkhead” or “winged-helix” DNA-binding domain,^[7]^ which is characterized by 100 amino acids in length and contains 3 α helices and 3 β folds. In 1998, the first FOX family member was identified in *Drosophila*. To date, hundreds of *FOX* genes have been identified in mammals, with 50 members in the human genome (plus 2 known pseudogenes *FOXO1B* and *FOXO3B*) and 44 members in mice, which can be divided into 19 subfamilies from FOXA to FOXS.^[7,8]^ The *FOX* gene family encodes proteins that play key roles in multiple biological processes (BPs) including metabolism, development, differentiation, proliferation, apoptosis, migration, invasion, and longevity.^[9]^ In recent years, accumulating studies reveal that FOX family members are involved in the occurrence and development of many cancers, including regulating the proliferation, migration, and invasion of tumor cells and affecting the tumor microenvironment (TME).^[10]^ Due to the large number of FOX families, we focused on the key components of 6 subfamilies in this study. Studies have found that FOXA1, FOXM1, FOXO3, FOXP1, and so on play an important role in tumors as oncogenes or tumor suppressor genes.^[11]^

FOXA1, a member of the FOX family of proteins, is a major regulator in hormone-sensitive tissues.^[12]^ Its expression is upregulated in a variety of cancers and is strongly correlated with tumor malignancy.^[13]^ In BRCA, FOXA1 is a favorable prognostic biomarker for patients with estrogen receptor (ER)-positive BRCA,^[14]^ and its upregulation is associated with good prognosis.^[15]^ And the upregulation of FOXA1 in ER-positive BRCA is the result of ER-dependent transcription regulation,^[16]^ which promotes BRCA progression while regulating the expression of tumor suppressor genes such as *E-cadherin* and *p27*.^[17]^ In addition, FOXC2 is highly expressed in BRCA and can be used independently as a prognostic marker for basal cell (BC)-like BRCA, and its overexpression promotes tumor growth, metastasis, and invasion by regulating epithelial-mesenchymal transition (EMT) in human BRCA cells.^[18]^ A study showed that FOXC2 mediates the G2/M transition in BRCA stem cell lines by upregulating the expression of cyclin-dependent kinase 1.^[19]^ FOXM1 is expressed in a variety of cancers,^[20]^ which can promote proliferation, migration, invasion, metastasis, and EMT of cancer cells.^[21]^ The miR-671-5P directly targets FOXM1 to inhibit its expression in BRCA, thereby suppressing proliferation, invasion, and EMT and inducing cell cycle arrest in the S phase.^[22]^ FOXO3 is a tumor suppressor that inhibits proliferation, tumorigenesis, and ER-mediated signaling in ER-positive BRCA cells.^[23]^ It has been found that *FOXO3* expression is downregulated in ER-positive BRCA s. The miR-940 can promote the proliferation and invasive ability of BRCA cells by targeting FOXO3.^[24]^ To date, FOXP1 has been identified as a tumor suppressor in several BRCA -related studies. FOXP1 enhances the migration of BRCA cells by inhibiting the transcriptional activity of NFAT1 in MDA-MB-231 cells.^[25]^ In recent years, FOXQ1 has been recognized as a proto-oncogene. The expression of FOXQ1 in BRCA is associated with poorer clinical prognosis^[26]^ and is involved in the regulation of EMT in BRCA cells.^[27]^ Recent studies have found that FOXQ1 promotes BRCA metastasis and progression by activating EMT transcription through the recruitment of mixed-lineage leukemia complexes.^[28]^

However, the underlying mechanisms of FOX transcription factor activation or repression and the different functions of FOX family members in BRCA have not been fully elucidated. In this study, the differential expression, mutation, function, prognostic significance, and immune infiltration of different FOX transcription factors in patients with BRCA were analyzed based on multiple large databases, which will help to further understand their potential role in BRCA.

## 2. Materials and methods

### 2.1. Differential expression of FOX transcription factor in carcinoma and paraneoplastic tissues

Using The Cancer Genome Atlas (TCGA) and Genotype-Tissue Expression (GTEx) TPM (transcripts per million reads) format, RNA sequencing (RNAseq) data are uniformly processed by the Toil process in UCSC XENA (https://xenabrowser.net/datapages/), and the RNAseq data in TPM format are converted into log2 data and then the R package ggplot2 (version 3.3.3) in R software (version 3.6.3, Vienna University of Economics and Business) is used to draw a differential expression map. Differences in transcript expression were compared using the Student *t* test, with *P* < .01 considered statistically significant.

### 2.2. GEPIA database analysis

The Gene Expression Profiling Interactive Analysis (GEPIA) (http://gepia.cancer-pku.cn/index.html) is a developed interactive web server for analyzing the RNAseq expression data based on tumor and normal samples from the TCGA and the GTEx databases,^[29]^ such as tumor/normal differential expression analysis, pathological stages, survival analysis, correlation analysis, and similar genes detection and dimensionality.^[30]^ In this study, GEPIA was used to analyze the differential gene expression, pathological stage, and correlative prognostic of tumor tissues and normal tissues. Student *t* test is used to generate a *P* value for the expression or pathological stage analysis. Differential expression of tumor and related normal tissues was analyzed by “analysis of variance.” Data were obtained from TCGA and GTEx databases. Filters were entered into the relevant data retrieval interface: |Log2FC|cutoff 1; *q* value 0.01. Comparative analyses were performed between cancer specimens and normal control data sets for each gene. At the same time, the similar gene detection module of the GEPIA database was used to screen out the 20 most similar genes of each member of the FOX family. After the intersection was removed, the top 106 genes significantly associated with FOX were provided in the enrichment analysis.

### 2.3. HPA database analysis

The Human Protein Atlas (HPA) (https://www.proteinatlas.org) is an information database of protein expression patterns for nearly 20 kinds of cancers.^[21]^ HPA applies transcriptome and proteomics to provide different protein profiles, including tissue maps, cell maps, and pathology maps. Users can identify tumor-type specific protein expression patterns that are differentially expressed in a given type of tumor. In this study, immunohistochemical images were used to directly compare the protein expression of different FOX family members in human normal tissues and BRCA tissues.

### 2.4. Kaplan–Meier plotter

Kaplan–Meier (K-M) plotter (http://kmplot.com/analysis/) is an integrated platform that can correlate gene expression with survival for 21 cancers, including bladder cancer, pancreatic cancer, liver cancer, BRCA, ovarian cancer, lung cancer, and gastric cancer.^[31,32]^ In our study, patients with cancer were divided into high- and low-expression groups based on the median *mRNA* expression value of each gene, and stratification was validated using a K-M plotter to analyze the different *FOX mRNA* expression and prognostic value of patients with BRCA. The prognostic value of the *mRNA* expression of distinct FOX in patients with BRCA was analyzed by using K-M plotter. Information on the number of risk cases, median *mRNA* expression levels, ratios, 95% confidence intervals, and *P* value can be found on the K-M plotter web page. When the *P* value < .05, the difference was statistically significant.

### 2.5. TCGA and cBioportal analysis

TCGA database is a publicly available database of tumor gene information, containing high-throughput sequencing and pathological data for 30 different cancers. A large number of cancer gene maps can be systematically analyzed to find out the similarities and differences in the genomic architecture of each cancer and across multiple types.^[32,33]^ cBioportal (www.cbioportal.org) is a comprehensive online open source that provides us with visualized and multidimensional cancer genomic data. The genetic alterations of FOX were obtained from cBioportal based on TCGA database. The association of *FOX* gene mutations with overall survival (OS), disease-free survival (DFS), and progression-free survival (PFS) of patients with BRCA was illustrated by K-M curves. A log-rank test was performed to determine the significant difference between the survival curves, with a *P* value < .05 indicating a statistically significant difference.

### 2.6. Construction of protein–protein interaction network

STRING (https://string-db.org/) is a database on protein–protein interactions (PPIs), which integrates experimental interaction evidence and computational interaction prediction information with the goal of achieving a comprehensive and objective global network.^[34]^ Different expressions and potential interactions of FOX and its similar genes were constructed by PPI network analysis. The PPI network constructed in the String database was then imported into Cytoscape (version: 3.9.1, National Resource for Network Biology) software for visualization.^[35,36]^ Finally, the degree algorithm in the CytoNCA plug-in was applied to sequence the genes.^[37]^

### 2.7. GeneMANIA analysis

GeneMANIA (http://www.genemania.org) is a resourceful database that uses large amounts of genomics and proteomics data to generate hypotheses about gene function, analyze gene lists, and prioritize genes for functional analysis.^[38]^ We use it to measure the predictive value of FOX and its related genes.

### 2.8. GO and KEGG enrichment analysis

Gene ontology (GO) and Kyoto Encyclopedia of Genes and Genomes (KEGG) enrichment analyses are performed by the DVAID database (https://david.ncifcrf.gov/). DVAID is a database for annotation, visualization, and integrated discovery designed to facilitate high-throughput gene functional analysis.^[39]^ The database integrates different public bioinformatics database resources and not only provides the typical gene-term enrichment analysis but also provides new tools and functions.^[40]^ The GO terminology consists of 3 categories: BPs, cellular components (CCs), and molecular functions (MFs). GO and KEGG analysis diagrams were drawn with the R package ggplot2 (version 3.3.3) in R software (version 3.6.3).

Gene sets with nominal *P* value < .05 and a false discovery rate < 0.25 were considered statistically significant.

### 2.9. TIMER analysis

Tumor Immune Estimation Resource (TIMER) is a user-friendly web interface for the comprehensive analysis of tumor-infiltrating immune cells (https://cistrome.shinyapps.io/timer/). It provides 6 main analysis modules for the systematic assessment of the infiltration of different immune cells and their clinical effects.^[36]^ Six types of immune cells (B cells, CD4 + T cells, CD8 + T cells, neutrophils, macrophages, and dendritic cells) were sequentially selected by entering FOX into the “Gene Module,” and scatter plots were generated to visualize the correlation between their expression and the degree of immune cell infiltration in BRCA.

## 3. Result

### 3.1. *mRNA* expression levels of different FOXs family members in human cancers

**Figure 1.**
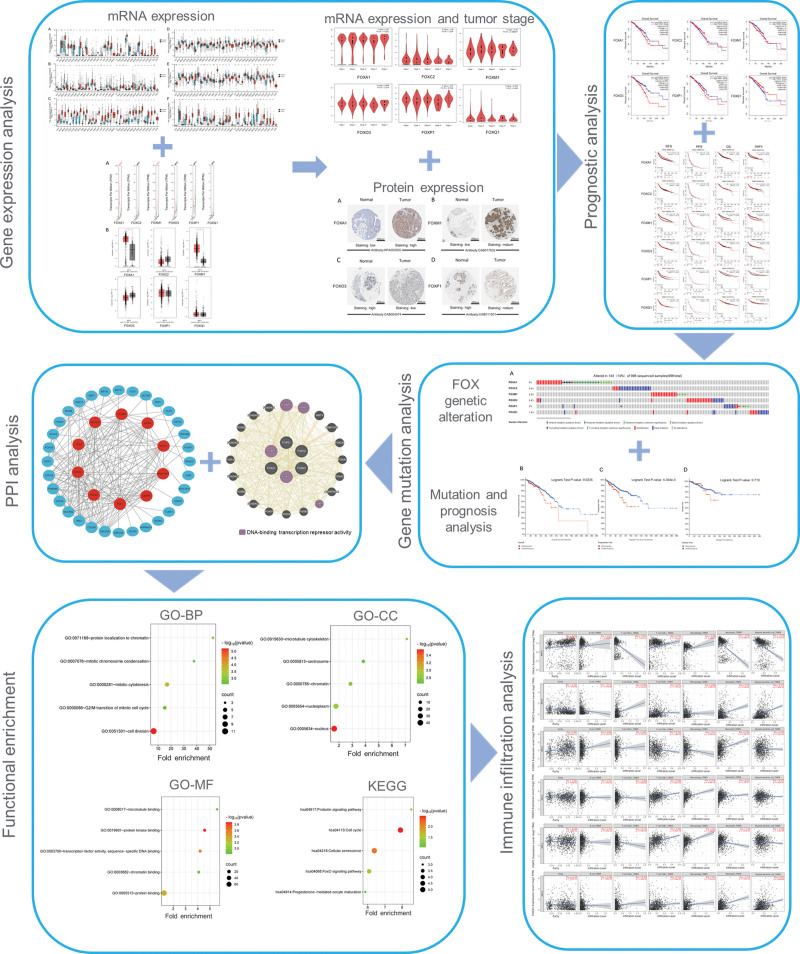
Flow chart of this study.

**Figure 2.**
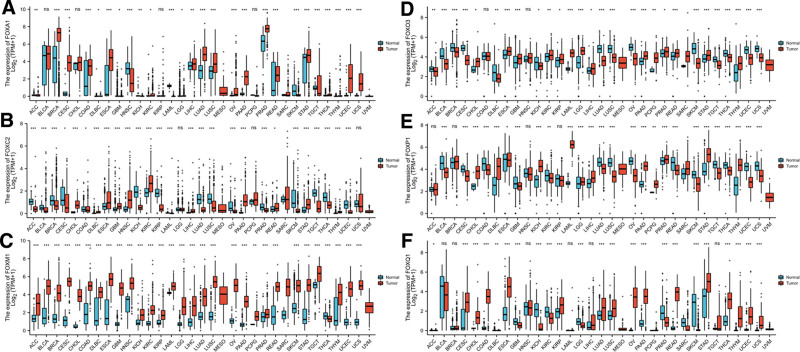
mRNA expression level of FOX transcription factor in different cancer types. As shown in figure, FOXA1 and FOXM1 mRNA were higher in breast cancer tissues than in precancerous tissues (*P* < .001, the difference was statistically significant). In contrast, FOXC2 and FOXO3 mRNA were significantly downregulated in breast cancer tissues than in precancerous tissues (*P* < .001, the difference was statistically significant). ns = *P* ≥ .05; *P < .05; **P < .01; ***P < .001. FOX = forkhead box.

The flowchart of this research is shown in Figure 1. The pan-cancer analyses were performed to compare the expression of FOX in the tumor samples of GTEx combined with TCGA and the corresponding normal samples of TCGA by Wilcoxon rank sum test. As shown in Figure 2, among the 33 cancer types, 26 types of analysis showed that the *mRNA* expression level of FOXA1 was significantly different between cancer tissue and precancerous tissue, among which 19 types showed upregulated expression and 7 types downregulated expression. FOXC2 was significantly upregulated in 11 analyses while downregulated in 17 analyses. Similar results were found for FOXO3, FOXP1, and FOXQ1. *FOXM1* expression increased in 29 groups of analyses and decreased only in thyroid carcinoma. In BRCA, FOXA1 and FOXM1 mRNA were higher in cancer tissues than in precancerous tissues (*P* < .001, the difference was statistically significant). Although the expressions of FOXP1 and FOXQ1 mRNA were increased in cancer tissue, the difference was not statistically significant (*P* > .05). In contrast, the expressions of FOXC2 and FOXO3 mRNA were significantly downregulated in cancer tissues (*P* < .001, the difference was statistically significant).

### 3.2. mRNA and protein expression levels of FOXs in BRCA

**Figure 3.**
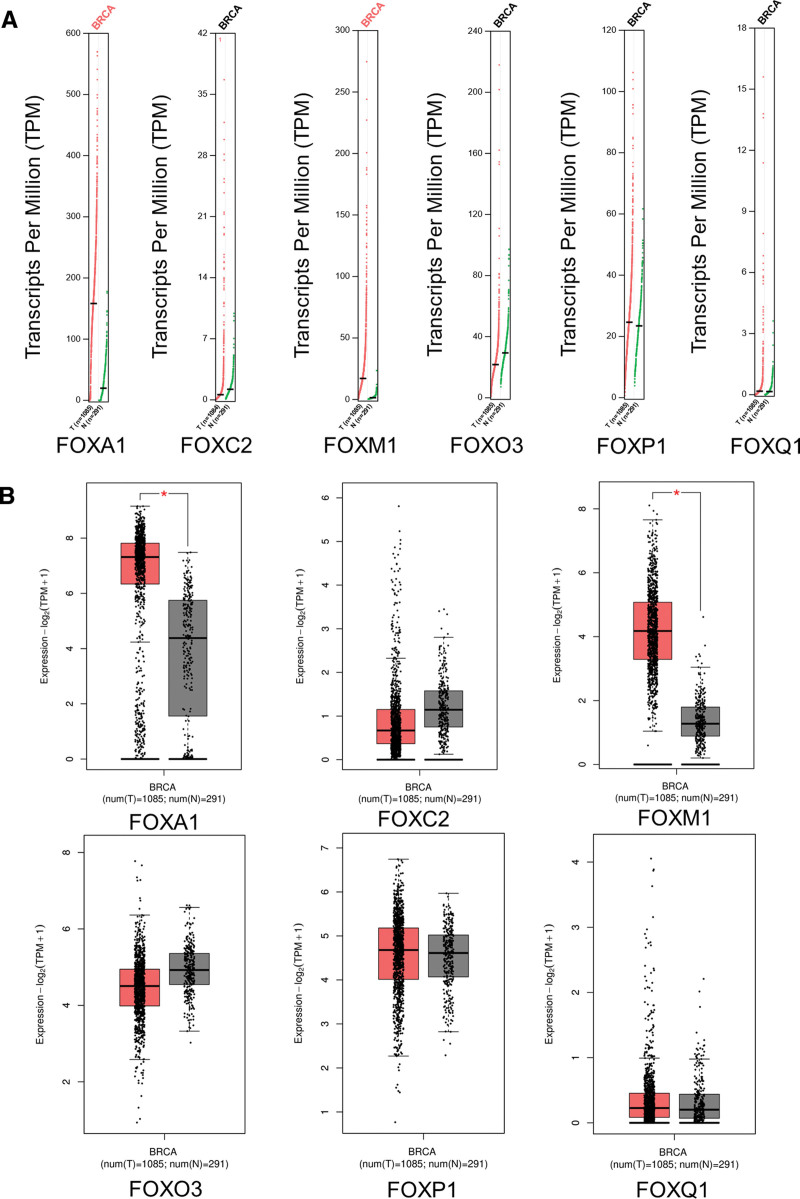
Expression of FOX transcription factor in breast cancer (GEPIA). (A) Scatter plot. (B) Block diagram. The expression levels of FOXA1 and FOXM1 in breast cancer tissues were significantly higher than those in normal tissues, while the expression levels of FOXC2 and FOXO3 in breast cancer tissues were lower than those in normal tissues. FOX = forkhead box, GEPIA = gene expression profile interaction analysis.

**Figure 4.**
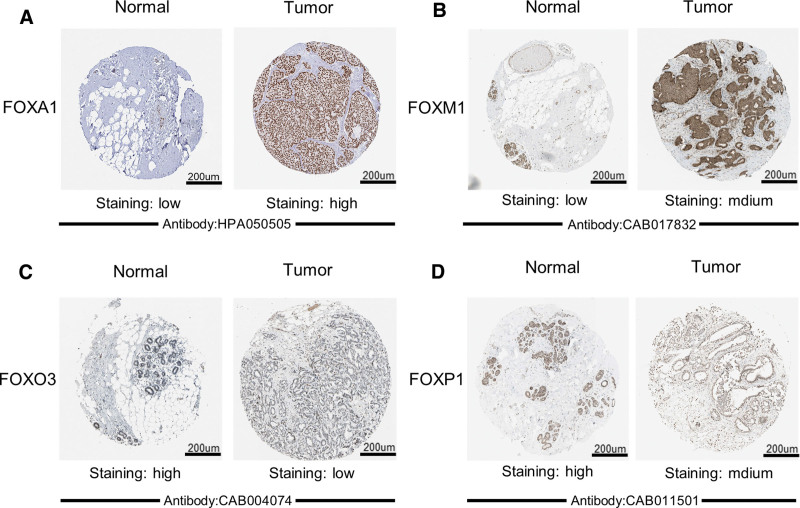
Representative immunohistochemistry images of FOX in breast cancer tissue and normal tissue (HPA). FOXA1 protein was not expressed in normal breast tissues, while it was highly expressed in breast cancer tissues, and FOXM1 protein had higher expression in breast cancer tissues than in normal tissues (A, B). In addition, FOXO3/FOXP1 was highly expressed in normal breast tissues, whereas it had low and moderate expression in breast cancer tissues (C, D). FOX = forkhead box, HPA = Human Protein Atlas.

Using the GEPIA data set (http://gepia.cancer-pku.cn/), we compared the *mRNA* expression of FOX factors between BRCA and normal tissues. The results indicated that the expression levels of FOXA1 and FOXM1 in BRCA tissues were significantly higher than those in normal tissues, while the expression levels of FOXC2 and FOXO3 in BRCA tissues were lower than those in normal tissues (Fig. 3). After examining the *mRNA* expression patterns of FOX in BRCA, we tried to explore the protein expression patterns of FOX in BRCA tissues and normal breast tissues using the HPA. As shown in Figure 4, with the exception of FOXC2 and FOXQ1 deletion information, FOXA1 protein is not expressed in normal breast tissue but is highly expressed in BRCA tissues, FOXM1 protein expression was higher in BRCA tissues than in normal tissues (Fig. 4A, B). In addition, FOXO3/FOXP1 was highly expressed in normal breast tissue, while it was low and moderately expressed in BRCA tissues (Fig. 4C, D). Taken together, our results showed that the transcriptional levels and protein expression of FOXA1/FOXM1 are upregulated in patients with BRCA.

### 3.3. Relationship between the expression levels of different FOXs family members and clinicopathological parameters in patients with BRCA

**Figure 5.**
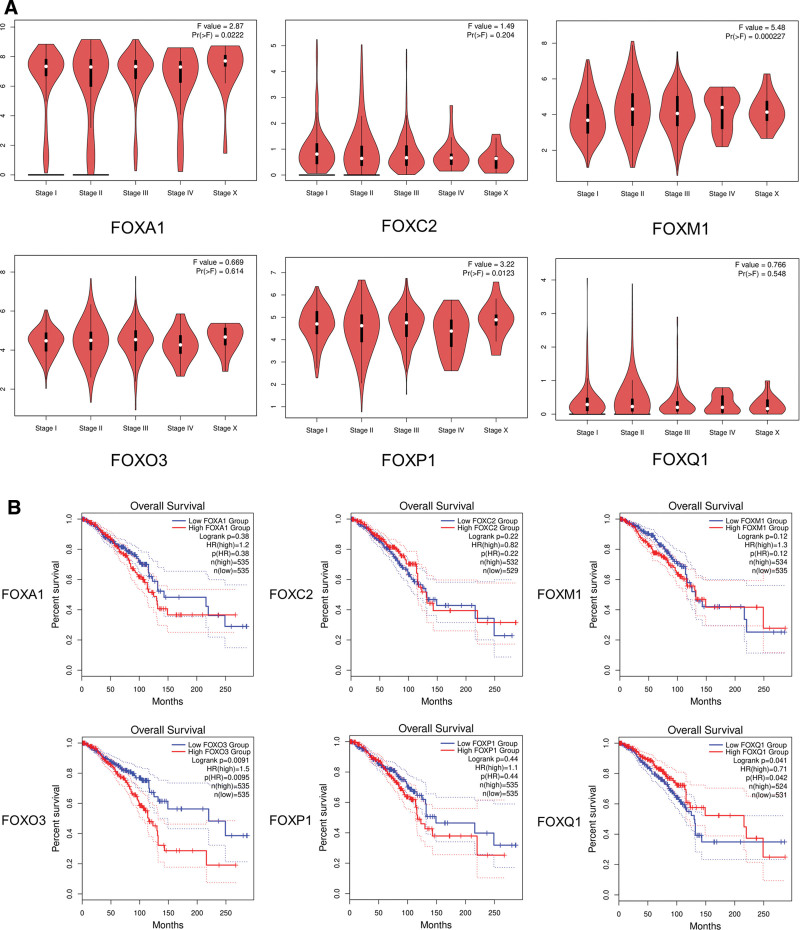
Relationship between clinical characteristics and FOXs. (A) Correlation between FOX expression and tumor stage in patients with breast cancer (GEPIA). The expression of FOXA1, FOXM1, and FOXP1 was correlated with the pathological stage of patients with breast cancer (*P* < .05). (B) Prognostic value of the mRNA expression of distinct FOX family members in breast cancer (GEPIA). Patients with breast cancer with high transcriptional levels of FOXO3 (*P* = .0091) were significantly associated with short OS, while patients with breast cancer with high transcriptional levels of FOXQ1 (*P* = .041) were significantly associated with longer OS. FOX = forkhead box, GEPIA = gene expression profile interaction analysis, OS = overall survival.

As shown in Figure 5A, we also analyzed the expression of FOX in BRCA tumor stages. Significant differences were found in groups FOXA1, FOXM1, and FOXP1, but not in groups FOXC2, FOXO3, and FOXQ1 (Fig. 5A). *FOXM1* gene expression is most significantly different in different stages of tumor development, and it is speculated that FOXM1 has prognostic significance in BRCA.

### 3.4. Expression and prognostic value of FOXs in patients with BRCA

To evaluate the value of differentially expressed FOX in BRCA, correlations between different FOX and clinical outcomes were analyzed using GEPIA. The relationship between the differential expression of FOX and OS in BRCA is presented in Figure 5B. Patients with BRCA with high transcriptional levels of FOXO3 (*P* = .0091) were significantly associated with short OS, while those with high transcriptional levels of FOXQ1 (*P* = .041) were significantly associated with longer OS. Except for FOXO3 and FOXQ1, other FOX family members did not seem to have a significant effect on OS. The K-M curve and log-rank test analyses revealed (Figure S1, Supplementary Digital Content, http://links.lww.com/MD/M34) that the recurrence-free survival (RFS) and DMFS of patients in the FOXA1 mRNA high-expression group were significantly better than those in the low-expression group (*P* < .05), while the expression of FOXA1 mRNA was not significantly related to postprogression survival (PPS) and OS. Contrary to FOXA1, RFS, OS, and DMFS in the high-expression FOXM1 group were worse than those in the low-expression FOXM1 group (*P* < .05). The increased FOXP1 mRNA level was significantly associated with RFS, OS, and DMFS in all patients with BRCA (*P* < .05). The results indicated that increased *FOXP1 mRNA* expression in patients with BRCA is associated with improved prognosis.

### 3.5. Gene mutation of FOXs in patients with BRCA and its association with OS, DFS, and PFS

**Figure 6.**
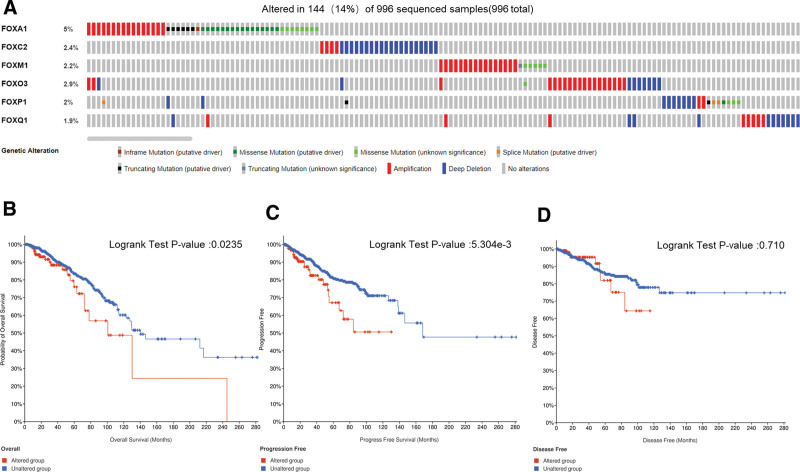
FOX gene mutation and association with OS, PFS, and DFS in patients with breast cancer (cBioPortal). (A) Mutation analysis of FOX gene in breast cancer. FOXs were altered in 144 samples of 996 patients with breast cancer, accounting for 14%. Relationship of FOX gene mutations to (B) OS, (C) PFS, and (D) DFS. Genetic alterations in FOX transcription factors were significantly associated with OS and PFS (B, C; P < .05), but not with DFS (D: P = .710) in patients with breast cancer. DFS, disease-free survival, FOX = forkhead box, OS = overall survival, PFS = progression-free survival.

cBioPortal was used to analyze the genetic variation of FOX family members and its effect on OS, DFS, and PFS in patients with BRCA. FOXs were altered in 144 samples of 996 patients with BRCA, accounting for 14%. In addition, FOXA1, FOXC2, FOXM1, FOXO3, FOXP1, and FOXQ1 were altered in 5%, 2.4%, 2.2%, 2.9%, 2%, and 1.9% of the queried BRCA samples, respectively (Fig. 6A). The results of K-M plotter and log-rank test indicated that the genetic change of FOXs was significantly correlated with OS and PFS of patients with BRCA (Fig. 6B, C; *P* < .05) but not with DFS of patients with BRCA (Fig. 6D; *P* = .710). These results suggest that genetic alterations of FOX transcription factors may significantly affect the prognosis of patients with BRCA.

### 3.6. Effects of FOX and closely similar genes on predictive function and pathways in patients with BRCA

**Figure 7.**
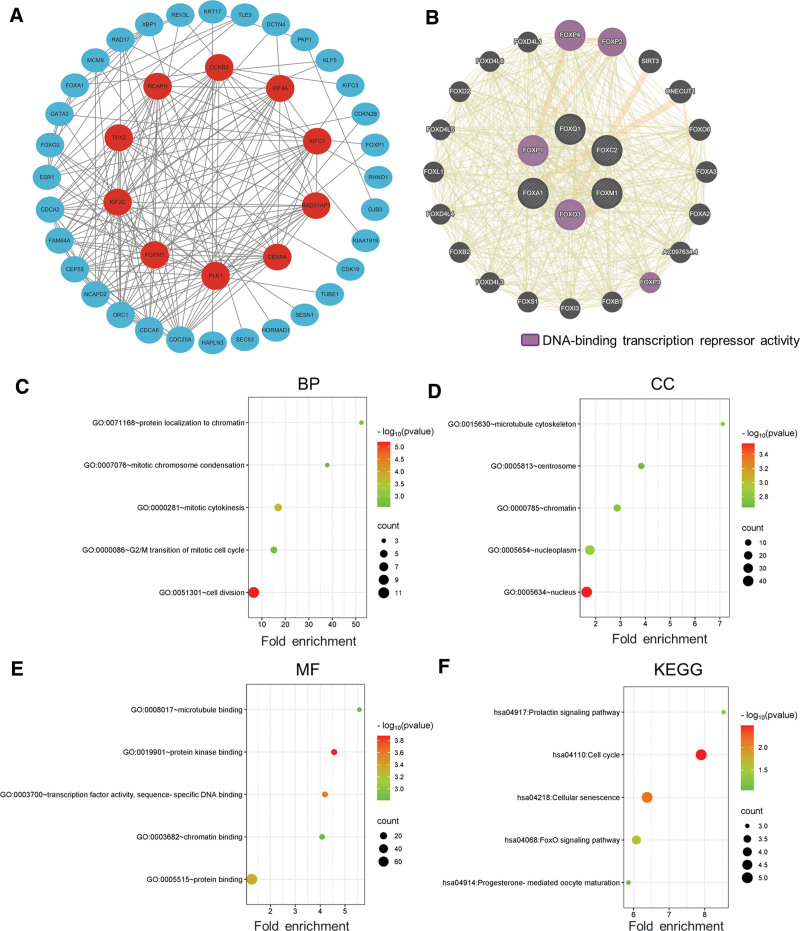
Enrichment analysis of FOX family members and their similar genes in breast cancer. (A) Protein–protein interaction (PPI) network of FOX and its similar genes. The network for FOX family members and 100 functionally similar genes. As expected, the 42 nodes and 179 edges were obtained in the PPI network. (B) The GeneMANIA results also revealed that the functions of FOXs and their similar molecules (such as FOXP4, FOXP2, SIRT3, ONECUT, FOXO6, FOXA3, FOXA2, and FOXP3) were primarily related to DNA-binding transcriptional inhibitor activity (GeneMANIA). (C–E) GO enrichment analysis results. (F) KEGG enrichment analysis results. FOX = forkhead box, GO = gene ontology, KEGG = Kyoto Encyclopedia of Genes and Genomes.

We used the similar gene detection module in GEPIA2 to obtain the 20 most similar genes for each FOX family member and a total of 106 genes were obtained after removing the intersection. Then, the STRING database was applied to construct a network analysis including FOX and its similar genes, and the network was imported into Cytoscape for visualization to explore their potential interactions. As expected, the 42 nodes and 179 edges were obtained in the PPI network (Fig. 7A). The results showed that the top 10 genes with the highest degree score were *PLK1, FOXM1, KIF4A, KIF2C, TPX2, NCAPH, CCNB2, CDC25A, CDCA8*, and *KIFC1*, and these genes were most associated with other genes in the network. The results of STRING database analysis showed that FOX and its similar genes are closely related to BPs of mitotic sister chromatid segregation, nuclear division, and epithelial cell development. The GeneMANIA results also revealed that the functions of FOXs and their similar molecules (such as FOXP4, FOXP2, SIRT3, ONECUT, FOXO6, FOXA3, FOXA2, and FOXP3) were primarily related to DNA-binding transcriptional inhibitor activity (Fig. 7B).

Enrichment analysis helps us to further understand the biological function of the FOX family in BRCA (Fig. 7C–F; Table 1). The function of FOX and similar genes was predicted by analyzing GO and the KEGG. GO enrichment analysis predicted the functional roles of genes on the basis of 3 aspects, including BPs, CCs, and MFs. The terms of BP mainly included GO:0051301 (cell division), GO:0000281 (mitotic cytokinesis), GO:0071168 (protein localization to chromatin), GO:0000086 (G2/M transformation of mitotic cell cycle), and GO:0007076 (mitotic chromosome condensation); the terms of CC mainly included GO:0005634 (nucleus), GO:0005654 (nucleoplasm), GO:0015630 (microtubule cytoskeleton), GO:0000785 (chromatin), and GO:0005813 (centrosome); and the terms of MF mainly included GO:0019901 (protein kinase binding), GO:0003700 (transcription factor activity, sequence-specific DNA binding), GO:0005515 (protein binding), GO:0003682 (chromatin binding), and GO:0008017 (microtubule binding) (Fig. 7A–C). As shown in Figure 7F and Table 1, 5 KEGG pathways are significantly associated with FOXs and their similar genes in BRCA: hsa04110 (cell cycle), hsa04218 (cellular senescence), hsa04068 (FOXO signaling pathway), hsa04917 (prolactin signaling pathway), and hsa04914 (progesterone-mediated oocyte maturation).

**Table 1:** The GO and KEGG function enrichment analysis.

ID	Category	Description	Count	%	*P* value	Fold enrichment
GO:0051301	BP	Cell division	11	11.957	6.28E−06	6.524
GO:0000281	BP	Mitotic cytokinesis	5	5.435	2.07E−04	16.952
GO:0071168	BP	Protein localization to chromatin	3	3.261	.001	52.420
GO:0000086	BP	G2/M transition of mitotic cell cycle	4	4.348	.002	15.144
GO:0007076	BP	Mitotic chromosome condensation	3	3.261	.003	37.859
GO:0005634	CC	Nucleus	42	45.652	2.78E−04	1.640
GO:0005654	CC	Nucleoplasm	30	32.609	.001	1.769
GO:0015630	CC	Microtubule cytoskeleton	6	6.522	.002	7.117
GO:0000785	CC	Chromatin	13	14.130	.002	2.869
GO:0005813	CC	Centrosome	9	9.783	.002	3.838
GO:0019901	MF	Protein kinase binding	11	11.957	1.32E−04	4.564
GO:0003700	MF	Transcription factor activity, sequence-specific DNA binding	11	11.957	2.61E−04	4.194
GO:0005515	MF	protein binding	74	80.435	4.49E−04	1.250
GO:0003682	MF	Chromatin binding	9	9.783	.002	4.077
GO:0008017	MF	Microtubule binding	7	7.609	.002	5.580
hsa04110	KEGG	Cell cycle	5	5.435	.003	7.902
hsa04218	KEGG	Cellular senescence	5	5.435	.007	6.382
hsa04068	KEGG	FOXO signaling pathway	4	4.348	.026	6.080
hsa04917	KEGG	Prolactin signaling pathway	3	3.261	.046	8.534
hsa04914	KEGG	Progesterone-mediated oocyte maturation	3	3.261	.089	5.857

Analysis of GO function enrichment and KEGG function enrichment of forkhead box (FOX) family members and 100 functionally similar genes in breast cancer.

BP = biological processes, CC = cellular component, GO = gene ontology, KEGG = Kyoto Encyclopedia of Genes and Genomes, MF = molecular function.

### 3.7. Correlation between *mRNA* expression of FOXs family members and immune infiltration level in patients with BRCA

**Figure 8.**
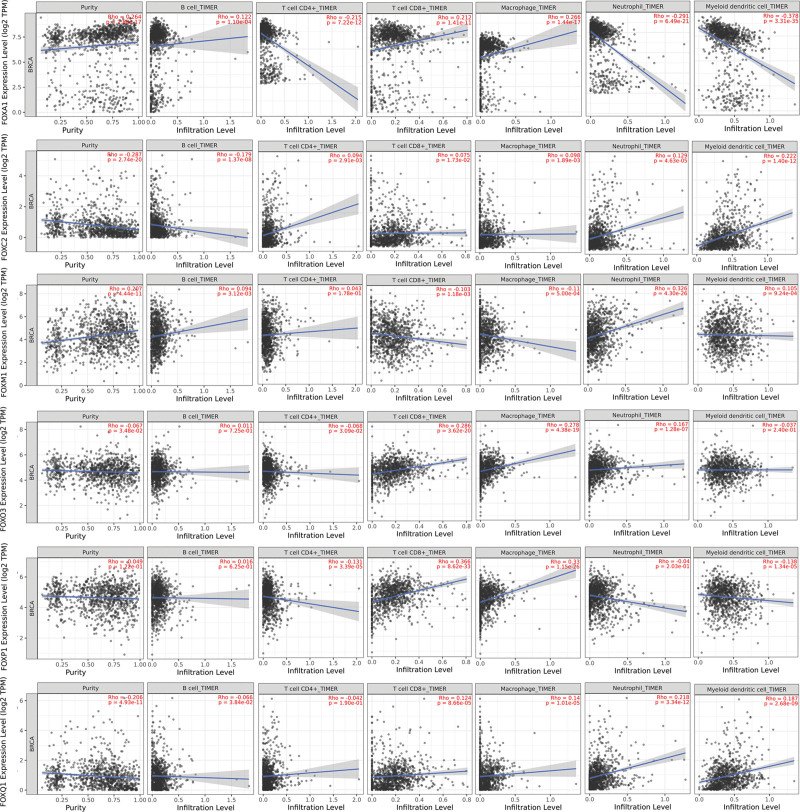
Correlation between *FOX mRNA* expression level and immune cell infiltration in breast cancer (TIMER). *FOXA1* expression was associated with the infiltration of CD4 + T cells, neutrophils, B cells, CD8 + T cells, macrophages, and dendritic cells in breast cancer tissues. *FOXC2* expression was negatively associated with the infiltration of B cells and positively associated with the infiltration of neutrophils and dendritic cells in breast cancer tissues. FOXM1 was negatively associated with the infiltration of CD8 + T cells, and macrophages and positively associated with the infiltration of neutrophils and dendritic cells. FOX = forkhead box, TIMER = Tumor Immune Estimation Resource.

In this study, the TIMER database was used to explore the correlation between FOX members and immune cell infiltration (Fig. 8). We found that FOXA1 and FOXM1 were significantly positively correlated with tumor purity in patients with BRCA, while FOXC2, FOXO3, FOXP1, and FOXQ1 were significantly negatively correlated with tumor purity in patients with BRCA. *FOXA1* expression is associated with the infiltration of CD4 + T cells, neutrophils, B cells, CD8 + T cells, macrophages, and dendritic cells in BRCA tissues. *FOXC2* expression was negatively associated with the infiltration of B cells in BRCA tissues and positively associated with the infiltration of neutrophils and dendritic cells. In patients with BRCA, *FOXM1* expression was negatively associated with the infiltration of CD8 + T cells and macrophages and positively associated with the infiltration of neutrophils and dendritic cells. In addition, infiltration of CD8 + T cells, macrophages, neutrophils, and dendritic cells in patients with BRCA was associated with *FOXO3* expression. *FOXP1* expression was negatively associated with the infiltration of CD4 + T cells and dendritic cells and positively associated with the infiltration of CD8 + T cells and macrophages. The expression of FOXQ1 was positively associated with the infiltration of CD8 + T cells, macrophages, neutrophils, and dendritic cells in BRCA. These results indicated that the FOX family may be involved in the immune infiltration process of BRCA cells.

## 4. Discussion

To date, a large number of studies have shown that the FOX transcription factor is involved in the occurrence and development of a variety of cancers, and the role of their abnormal expression in a variety of cancers has been reported. However, the different roles of FOX transcription factor family members in BRCA remain to be further analyzed. In this study, we attempted to comprehensively analyze different FOX transcription factors in BRCA in terms of expression, mutation, prognostic value, functional enrichment, and immune cell infiltration.

FOXA1 is expressed in all luminal BRCA cell lines, all ER-positive tumors, and about half of ER-negative tumors.^[41]^ In ER-positive BRCA, FOXA1 is a positive prognostic factor,^[15]^ and its high expression positively correlates with the ductal A subtype.^[14]^ FOXA1 has been reported to be a key determinant of ER function and endocrine response,^[42]^ mediating endocrine resistance by altering the ER transcriptome and IL-8 expression.^[10,43]^ In our study, TCGA combined with GTEx data and gene expression profile data set showed that the expression level of FOXA1 was significantly higher in BRCA tissue than in normal tissue, and the expression of FOXA1 was significantly associated with tumor stage (*P* < .05). In addition, among all patients with BRCA, the RFS and DMFS were significantly better in the FOXA1 mRNA high-expression group than in the low-expression group. These results illustrate that FOXA1 plays the role of a tumor suppressor gene in BRCA.

FOXC2 plays an important role in the carcinogenic process of a variety of cancers^[44]^ and promotes tumor metastasis by promoting cell proliferation, EMT, angiogenesis, and lymphangion genesis.^[9]^ High *FOXC2* expression was associated with aggressive basal-like BRCA subtypes.^[45]^ Studies have shown that *FOXC2* expression is upregulated in BRCA and can be independently used as a prognostic marker for basal-like BRCA. The researchers showed that FOXC2 mediates the G2/M transition in BRCA stem cell lines through upregulation of cell cycle protein-dependent kinase 1 (cyclin-dependent kinase 1) expression.^[19]^ In our study, *FOXC2* expression was lower in BRCA tissues than in normal tissues, but its expression was not significantly correlated with tumor stage. We guessed that the reason for this result was the sample size is too small, and the exact roles of FOXC2 in BRCA need further evaluation.

FOXM1 is one of the most intensively studied FOX transcription factor in cancer research. It is upregulated in a variety of cancers and plays an important role in the poor prognosis of malignant tumors. Studies have shown that FOXM1 can promote the proliferation, migration, invasion, metastasis, and epithelial-mesenchymal transformation of cancer cells.^[10]^ It is expressed in all subtypes of BRCA and is the factor most associated with the risk of poor survival, especially in triple-negative BRCA.^[46]^ The high expression of FOXM1 in tumors is associated with the activation of cancer-promoting signaling pathway and the stimulation of reactive oxygen species,^[47]^ but the high expression of miR-671-5P can directly target FOXM1 to inhibit its expression in BRCA.^[22]^ In our study, the expression of FOXM1 was significantly higher in BRCA tissues than in normal tissues, and this expression was significantly associated with tumor stage (*P* < .001). These results illustrate that FOXM1 has prognostic significance in cancer. In addition, the high expression of FOXM1 was significantly associated with low OS, RFS, and DMFS (*P* < .05), which seems to be consistent with the role of FOXM1 as an oncogene.

FOXO3 transcription factor was considered a tumor suppressor gene, which can inhibit cell proliferation and tumorigenesis.^[10]^ FOXO3 protein expression was negatively correlated with KI67 and markers of Phosphatidylinositol 3-kinase /AKT/mammalian target of the rapamycin oncogenic pathway activity and positively correlated with the apoptosis marker p53.^[48]^ Studies have found that *FOXO3* expression is downregulated in ER-positive BRCA, and its low expression is associated with poor prognosis of BRCA.^[23]^ In addition, FOXO3 can act as a protective agent to protect genomic stability and the health of hematopoietic stem cells from oxidative DNA damage.^[49]^ In our study, FOXO3 was significantly downregulated in BRCA tissues, but its expression was not significantly correlated with tumor stage in patients with BRCA. In all patients with BRCA, decreased *FOXO3* expression was associated with poor RFS, PPS, and DMFS, but there was no significant correlation.

FOXP1 has been identified as a tumor suppressor in several BRCA-related studies. Studies have shown that FOXP1 plays a key role in estrogen signaling and the biology of ERα-positive BRCA, and it can promote cancer cell proliferation by enhancing estrogen response element-mediated transcription.^[50]^ FOXP1 regulates the expression of chemokines and cytokines, which is an important negative regulator of immune invasion in BRCA.^[51]^ Some scholars have pointed out that FOXP1 may be a potential biomarker for the prognosis of BRCA.^[52]^ Its expression was associated with ERα, ERβ, and improved survival in familial BRCA.^[53]^ Similar to our study, the expression of FOXP1 in BRCA tissues was increased but not significant (*P* > .05). However, the results showed that the expression of FOXP1 was significantly correlated with the stage of BRCA. In all patients with BRCA, increased FOXP1 mRNA levels were significantly associated with RFS, OS, and DMFS of patients with BRCA (*P* < .05). This appears to be consistent with the role of FOXP1 as a tumor suppressor.

The study found that FOXQ1 is a direct transcriptional regulator of IL-1α, IL-8, and vascular endothelial growth factor in BRCA cells.^[54]^ FOXQ1 is upregulated in BRCA tissues, which promotes EMT, cell migration and invasion, and self-renewal and metastasis of BRCA stem cells.^[55]^ According to reports, the hypothesis that FOXQ1 is an independent predictor of OS in patients with BRCA was further demonstrated, and its low expression was associated with poor prognosis.^[56]^ In addition, a recent study showed that *FOXQ1* expression in BC cells is negatively regulated by FOXF2.^[57]^ In this report, we found that FOXQ1 mRNA expression was increased in BRCA tissues, but its expression was not associated with the tumor stage of BRCA. In all patients with BRCA, high *FOXQ1* expression was associated with poor RFS, PPS, and DMFS, but not significantly. And high *FOXQ1* expression was associated with longer OS. The role of FOXQ1 in BRCA needs further investigation.

We further analyzed the genetic variation in individual genes of the FOX transcription factor family and found that FOX is frequently genetically altered in BRCA. Genetic alterations in FOX transcription factors were significantly associated with shorter OS and PFS in patients with BRCA (Fig. 6B, C; *P* < .05) but not associated with DFS in patients with BRCA (Fig. 6D; *P* = .710). The GO and KEGG enrichment analysis of the FOX family and its functionally similar genes is helpful to accurately understand the cellular functions and related pathways of FOX involved in BRCA. Our study showed that these genes are mainly involved in cell division, cell senescence, cell cycle, and prolactin signaling pathway.

In recent years, the TME has been increasingly researched and has become an important therapeutic target.^[43]^ TME can promote tumor development, growth, and suppress immune function through a variety of complex intercellular signaling pathways.^[58]^ In our study, the expression levels of FOXA1, FOXC2, FOXO3, FOXM1, FOXP1, and FOXQ1 were significantly associated with the levels of immune cells. Among them, the expression of FOXA1 is associated with the infiltration of CD4 + T cells, neutrophils, B cells, CD8 + T cells, macrophages, and dendritic cells in BRCA tissues. However, FOXM1 is negatively associated with the infiltration of CD8 + T cells and macrophages, and positively associated with the infiltration of neutrophils and dendritic cells. These results suggest that these transcription factors may be involved in the immune infiltration process of BRCA cells, which provides a new research direction for molecular biomarkers, new treatment, and prognosis prediction of BRCA.

In this study, we systematically analyzed the expression and prognostic value of FOX in BRCA and provided insights into the heterogeneity and complexity of the molecular biological properties of BRCA. Our study provides multiple evidence for FOX family members as molecular markers for BRCA. Increased expression of FOXA1 and FOXM1 in BRCA tissues may play an important role in BC tumorigenesis. Highly expressed FOXA1, FOXM1, and FOXP1 may play an important role in BC tumorigenesis and can be used as molecular markers to identify BRCA stage and prognosis. However, there are certain limitations of our study. First, the data were downloaded from public databases and online websites, and the amount of data and information was limited and unbalanced, thus further cellular experiments and clinical studies are needed to confirm our findings to promote FOX as a diagnostic marker or therapeutic target for BRCA. Second, BRCA exhibits strong heterogeneity, and the mRNA expression levels in the TCGA database are the average mRNA expression levels of all cell types within the tumor, requiring single-cell sequencing to further elucidate the role of FOX in BRCA. Finally, the study only identifies associations and not causal relationships between FOX factors and outcomes.

## 5. Conclusions

In this study, we analyzed the expression, mutation, function, prognostic value, and infiltration of members of the FOX transcription factor family in BRCA using professional and reliable data. Our results showed that the expression levels of FOXA1 and FOXM1 are significantly elevated in BRCA tissues, while the expression levels of FOXC2 and FOXO3 were reduced. In addition, the high expression of mRNA in the FOXA1, FOXM1, and FOXP1 was significantly associated with tumor stage in BRCA tissues. The high expression of FOXM1 and FOXP1 was significantly associated with low OS, RFS, and DMFS, while the low expression of FOXA1 was significantly associated with low RFS and DMFS. These results indicate that FOXA1, FOXM1, and FOXP1 play an important role in the occurrence and development of BRCA and can be used as potential biomarkers for the prognosis of patients with BRCA. In addition, FOX is mainly involved in cell division, cell senescence, and cell cycle and may be involved in the prolactin signaling pathway. Then, we found that the expression of FOX transcription factors is closely associated with the infiltration of multiple immune cells. Our study provides novel insights for the selection of prognostic biomarkers of FOX family in BRCA and lays a foundation for further research on the immune infiltration role of FOX transcription factor family members in tumors.

## Author contributions

**Formal analysis:** Hui Yuan.

**Software:** Hui Yuan, Yu Liang.

**Visualization:** Hui Yuan.

**Writing—original draft:** Hui Yuan.

**Data curation:** Yu Liang, Jinxiang Chen, Min Zeng.

**Investigation:** Yu Liang, Jun Jiang.

**Resources:** Shaorun Hu, Jinxiang Chen, Jingcan You, Jun Jiang.

**Supervision:** Shaorun Hu, Jingcan You.

**Validation:** Shaorun Hu, Jinxiang Chen, Jun Jiang, Mao Luo.

**Funding acquisition:** Mao Luo, Min Zeng.

**Writing—review and editing:** Mao Luo.

**Conceptualization:** Min Zeng.

## Supplementary Material


