# Supplementary material for: The role of transcription factor FOXA1/C2/M1/O3/P1/Q1 in breast cancer
Source: Medicine (Baltimore). 2024 Apr 12;103(15):e37709. doi: 10.1097/MD.0000000000037709 (PMC11018205; doi:10.1097/MD.0000000000037709)
Supplement: Supplementary file 1 [file medi-103-e37709-s001.docx]

**The Role of transcription factor FOXA1/C2/M1/O3/P1/Q1 in breast cancer**

Hui Yuan^1^^, 2#^, Yu Liang ^3#^, Shaorun Hu ^2, 4, 5^, Jinxiang Chen ^2, 4, 5^, Jingcan You^2, 4, 5^, Jun Jiang^6,7^, Mao Luo ^2, 4, 5^*, Min Zeng ^1^*

Supplementary Material

**Supplementary Figure S1**


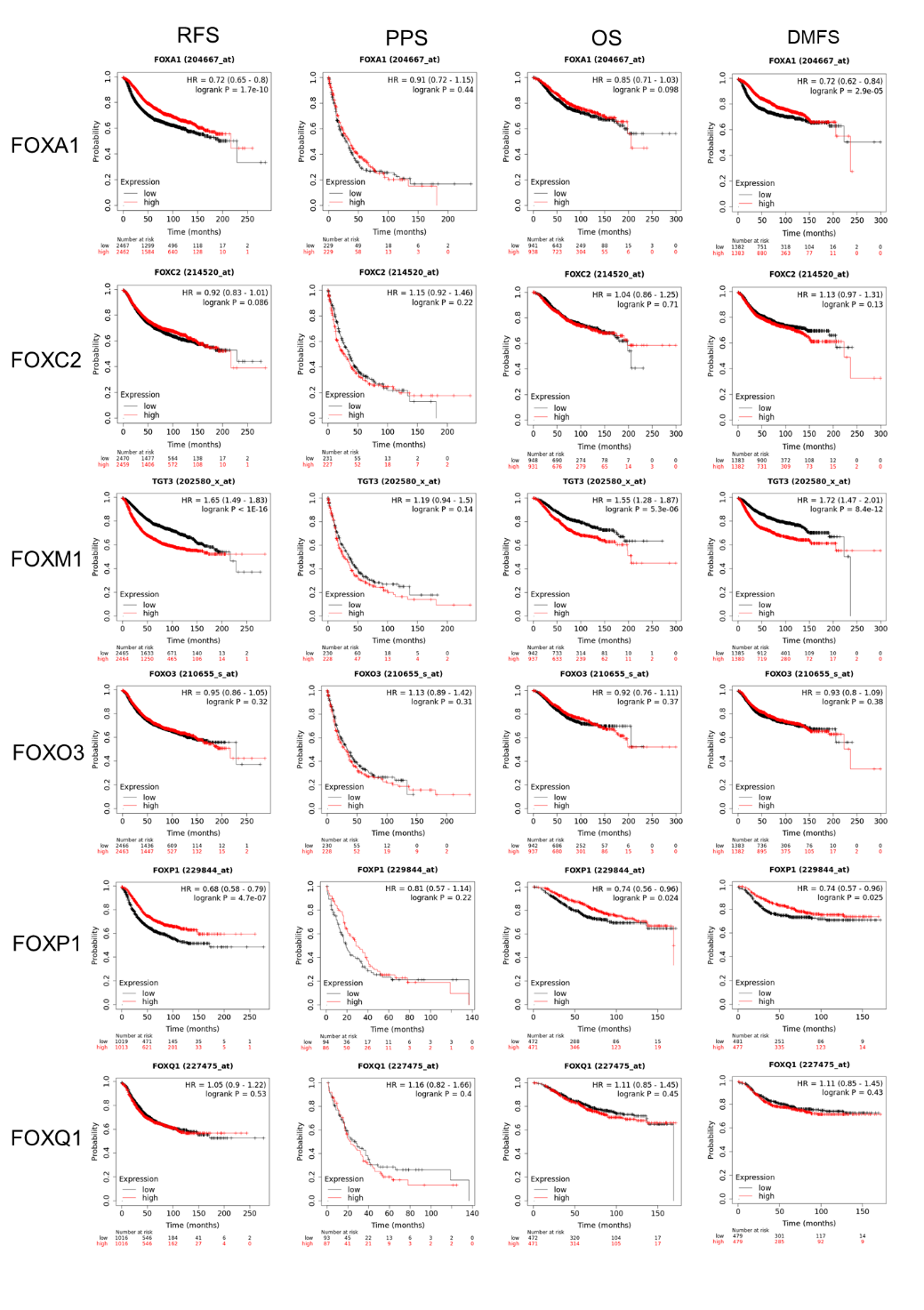


**Supplementary Figure S1. Prognostic value of FOX in breast cancer patients (K-M Plotter).** The Kaplan-Meier curve and log rank test analyses revealed that the RFS and DMFS of patients in the FOXA1 mRNA high-expression group were significantly better than those in the low-expression group (p<0.05), while the RFS, OS and DMFS in high-expression FOXM1 group were worse than those in low-expression FOXM1 group (p < 0.05). The increased FOXP1 mRNA level was significantly associated with RFS, OS and DMFS in all breast cancer patients (p<0.05).
